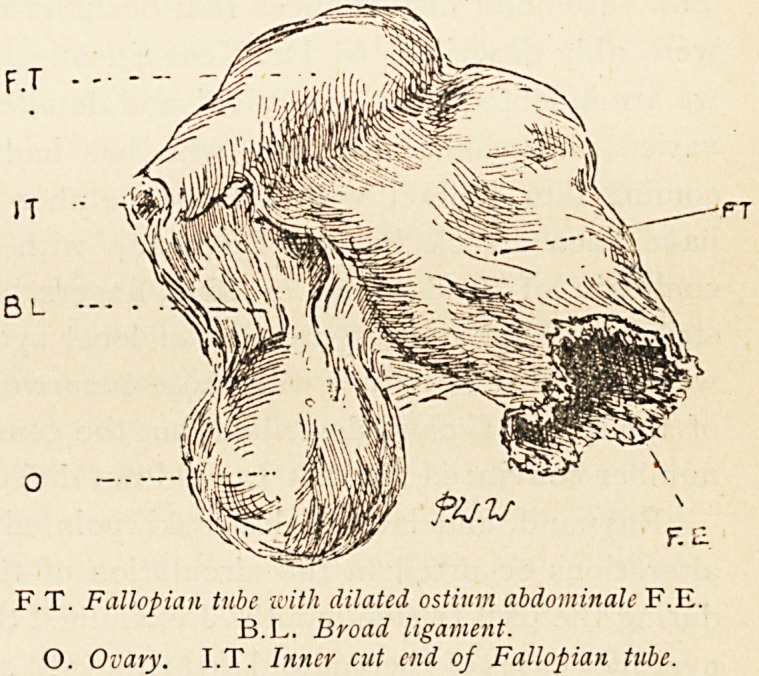# Tubal Abortion
^1^Paper read at the Meeting of the Bath and Bristol Branch of the British Medical Association, October 29th, 1902.


**Published:** 1902-12

**Authors:** D. C. Rayner

**Affiliations:** Assistant Obstetric Physician to the Bristol General Hospital


### TUBAL ABORTION.[1]

BY

D. C. Rayner, F.R.C.S.,

*Assistant Obstetric Physician to the Bristol General Hospital*

During the last few years considerable advance has been made in our knowledge of the early changes that take place in a gravid Fallopian tube. We know that when the ovum continues to develop until the tenth or twelfth week, the case usually terminates in rupture of the tube, with its well-known symptoms. But at an earlier period than this, during the first five or six weeks, other changes may take place, involving both the developing ovum and the tube in which it is contained. Of these changes, one of the most important and interesting is that of tubal abortion. We may, I think, define tubal abortion as the expulsion of an impregnated ovum into the peritoneal cavity through the ostium abdominale of an unruptured Fallopian tube. We are rather inclined to imagine that an extrauterine pregnancy usually terminates in rupture of the tube, whereas it is

[1] Paper read at the Meeting of the Bath and Bristol Branch of the British Medical Association, October 29th, 1902.

probable that, compared with "tubal abortion," rupture of the tube is a comparatively rare event. W. J. Taylor says that out of 21 cases of intraperitoneal hæmatocele due to tubal pregnancy, operated upon by himself, and in which he was able to examine the condition of the tube, 14 were due to hemorrhage from an unruptured tube, while only seven were associated with rupture; and Cullingworth's figures are still more striking; he says: "Of 25 cases of pelvic hæmatocele, in which an opportunity occurred of verifying by actual inspection the source of the bleeding, 23 were instances of hemorrhage from the open abdominal ostium of a pregnant Fallopian tube, and only two were due to rupture." During the present year, I have had two of these cases under my care, in one of which I opened the abdomen and removed the unruptured gravid tube. Of course we must not forget the possibility of an intra-pelvic hæmatocele with an unruptured tube being due to a primary ovarium pregnancy, several undoubted cases of which have now been recorded.

In order to understand the pathology of tubal abortion, it will be necessary to describe briefly the changes that take place both in the ovum and in the tube, previous to its occurrence. The changes that take place in the tube:—During the first month or six weeks that portion of the tube in which the ovum is lodged becomes very vascular and turgid, and as the tube expands from the growth of the fœtus and its membranes, the tubal mucous membrane is stretched and its folds effaced. Whether an actual decidual membrane is formed in the tube is from recent investigation rather doubtful. The placental site is represented only by a pseudo-decidual layer, composed of fibrin and connective tissue, so that the surroundings of the ovum are anything but favourable to its continued development. While these changes are taking place in the tube itself, others equally important are occurring at the abdominal ostium, which gradually bring about its occlusion. Careful observation, however, shows that closure of the abdominal ostium is by no means a constant sequel of tubal gestation, and it is in those cases in which the ostium remains patent that tubal abortion is especially liable to occur. The changes that take place in the developing ovum are of great interest and importance. We know that when the ovum develops in the uterus it is liable to a curious change, whereby it is converted into what is known as a mole. Now precisely the same thing may occur when the ovum develops in the tube; and just as the presence of a mole in the uterine cavity usually excites uterine contraction which end in its expulsion, so the presence of a mole in the outer portion of the tube excites contractions of the tube which result in its expulsion into the peritoneal cavity, and to this event the term tubal abortion is applied. But there is a still further resemblance between uterine and tubal abortion, for in both cases the abortion may be complete or incomplete. If it is complete the hemorrhage usually ceases; if, however, any portion of the ovum is retained, the hemorrhage from the tube persists, just as it would do from the uterus in a case of incomplete uterine abortion.

The symptoms of a tubal abortion, although in many cases quite characteristic, are in others very obscure. In a typical case the patient often after a period of sterility goes ten days or a fortnight over her period, when she is suddenly seized with (severe) pain in the lower part of the abdomen, with signs of internal hemorrhage. With or soon after the onset of the pain there is usually a discharge of blood from the uterus, and sometimes a decidual membrane is thrown off. The symptoms, however, are never so severe as in rupture of the tube, and in some cases the patient thinks it is nothing more than a delayed period, or an early miscarriage. The subsequent symptoms depend to a great extent upon whether the "tubal abortion" is of the complete or incomplete variety. If "complete," the hemorrhage from the tube usually ceases, and a small pelvic hæmatocele forms, which is gradually absorbed. If, however, the "abortion" is incomplete, the attacks of abdominal pain recur, the hemorrhage from the tube continues, and if active treatment is not undertaken the patient may bleed to death. On abdominal examination there is often nothing to be made out beyond some tenderness on palpation over the lower part of the abdomen. On vaginal examination, however, the signs are fairly characteristic. Soon after the onset we find a distinct swelling in Douglas's pouch, behind and to one side of the uterus, the swelling being due to the coagulated blood that has escaped from the open end of the tube and formed a hæmatocele there. We shall also find that as a rule this swelling increases in size with each attack of pain. Time will not allow me to discuss fully the differential diagnosis of tubal abortion, but I should like to draw attention to one condition which is especially liable to be mistaken for it, and that is an ordinary uterine abortion. These are the cases of tubal pregnancy which are sometimes mistaken for an ordinary miscarriage, and we see that the two conditions have many points in common. In both cases the patient has usually missed a period, and is suddenly seized with abdominal pain and uterine hemorrhage. A chief source of error in making a correct diagnosis is that in considering the symptoms, the possibility of an extra-uterine pregnancy is overlooked, and consequently a careful bimanual examination is not made. If this were done the presence of a mass in Douglas's pouch would go far to clear up the doubt. Considerable stress has been laid by some on the importance of examining the uterine discharge for chorionic villi in any doubtful case; of course a positive result would be diagnostic of intrauterine pregnancy, but a negative result would not necessarily mean that the pregnancy was extra-uterine, as the villi are sometimes very difficult to detect, especially in cases where the decidual membrane is disintegrated before it is thrown off. The diagnosis, however, between these two conditions is of the utmost importance as regards treatment, for should we be tempted to explore the uterine cavity in a case of tubal abortion, on the supposition that it was an ordinary miscarriage, the result might be disastrous.

With regard to the treatment of "tubal abortion," there is still some difference of opinion, especially as to when active surgical interference should be undertaken. The difficulty lies in our not being able to tell at once whether it is a case of complete or incomplete tubal abortion. Each case must be carefully studied and kept under constant observation; if the symptoms have never been severe, and tend to progressively improve under appropriate management, an expectant plan of treatment is indicated; if, on the other hand, the hæmatocele becomes larger and the general condition deteriorates, active treatment is necessary. Operative procedure, which in these cases consists in removal of the affected tube, may be carried out by the abdominal or vaginal route. The former is, I think, preferable, as it allows greater facility for examining the part and dealing with the damaged tube. In conclusion, I give very briefly the notes of a case of tubal abortion, which was under my care this summer, in which I removed the ovary and tube.

Patient, 34 years of age, had had three children; no miscarriages. Youngest child $6\frac{1}{2}$ years of age. Periods since last child have been regular every 28 days. For three weeks previous to her admission into the Hospital, the patient had been suffering from irregular uterine hemorrhage, after having gone eight days over her period. With the hemorrhage she had occasional attacks of abdominal pain; these, however, were not sufficiently severe to compel her to remain in bed. She herself thought she might have had an early miscarriage. The day before her admission into the Hospital (this was three weeks after the first attack of hemorrhage), she was seized with severe abdominal pain, and fainted; she rallied however, and seemed fairly comfortable, but during the night she had another attack, and Dr. Hedley Hill, who had seen her the previous afternoon, was fetched at 2.0 a.m., when he found her quite blanched and almost pulseless. He diagnosed a ruptured tubal pregnancy, and the same day sent her into the Hospital under me. When I saw her in the afternoon she had rallied wonderfully; her pulse was only 85, strong and full. On abdominal examination, there was some fulness and tenderness just above the pubes and Poupart's ligament on right side. On vaginal examination, a mass was felt in Douglas's pouch, pushing the cervix forward close behind the pubes. She complained of no pain, and felt very comfortable. I saw her again in the evening, when her condition was still very good, pulse being only 90. As, however, I thought the swelling in Douglas's pouch had increased, I decided to open the abdomen, when I found the pelvis and lower abdomen filled with blood-clot, the right tube distended to the size of a piece of small intestine, and a large clot

F.T. *Fallopian tube with dilated ostium abdominale* F.E. B.L. *Broad ligament.* O. *Ovary.* I.T. *Inner cut end of Fallopian tube.*

protruding from the dilated abdominal ostium. I removed the right tube and ovary, washed out the blood-clot with hot saline fluid, and closed the abdomen without drainage. The patient made a very good recovery. On examining the specimen there is no rupture of the tube to be seen, but its outer portion is much distended, and the ostium abdominale will admit the tip of the little finger. There is a corpus luteum in the ovary. An interesting feature of the case is, that for three weeks the symptoms closely resembled those met with in a case of incomplete uterine abortion, viz., persistent uterine hemorrhage, with occasional attacks of abdominal pain.

## Figures and Tables

**Figure f1:**